# Mentalising and social problem solving in adults with Asperger's syndrome

**DOI:** 10.1080/13546805.2013.809659

**Published:** 2013-07-22

**Authors:** Shelley Channon, Sarah Crawford, Danuta Orlowska, Nimmi Parikh, Patrizia Thoma

**Affiliations:** a Department of Cognitive, Perceptual and Brain Sciences, University College London, London, UK

**Keywords:** social cognition, problem-solving, mentalising, theory of mind, autism, Asperger's syndrome

## Abstract

**Introduction:**

It is well established that autistic spectrum disorder is linked to difficulties with mentalising, but the ways in which this affects everyday behaviour is less well understood. This study explored the nature and extent of difficulties in everyday social functioning in adults with Asperger's syndrome (AS), since increased understanding can enhance the development of more effective intervention strategies.

**Methods:**

Individuals with AS (*n* = 21) were compared with healthy control participants (*n* = 21) on three tests of social cognition: the Mentalistic Interpretation task, which assesses interpretation of sarcasm and actions; the Social Problem Fluency task, which assesses ability to generate problem solutions; and the Social Problem Resolution task, which assesses judgement in selecting problem solutions.

**Results:**

Comprehension of both sarcastic remarks and actions was impaired in those with AS on the mentalistic interpretation task. Participants with AS showed difficulties in identifying the awkward elements of everyday social scenarios, and they were also impaired in generating problem solutions but not in judging alternative solutions on the social problem fluency and resolution tasks.

**Conclusions:**

These tasks potentially provide a means of profiling strengths and weaknesses in social processing, which in turn has implications for informing clinical evaluation and training.

## Introduction

Positive social relationships exert a strong influence on people's physical (Cohen, Doyle, Skoner, Rabin, & Gwaltney, 1997) and psychological (Segrin & Taylor, 2007) health. An important factor in successful engagement in the social world is the ability to mentalise, i.e., to reason about others’ mental states. Difficulties in mentalising are thought to form the key impairment in individuals with autistic spectrum disorder (ASD; e.g., Frith, 2001). How these difficulties affect everyday behaviour is, however, less well understood. The focus of the present study was to explore this in more detail, since greater clarity about the impact on everyday behaviour can enable a wider range of intervention strategies to be developed.

It has been established that children and adults with Asperger's syndrome (AS) and high-functioning autism (HFA) sometimes pass both first-order belief tasks (involving inference that a story character's beliefs are diverging with reality) and second-order false belief tasks (involving inference about someone's false attribution of belief) (e.g., Bowler, 1992; Ozonoff, Pennington, & Rogers, 1991) despite the presence of significant difficulties in everyday social interactions (e.g., Frith, Happé, & Siddons, 1994). It is also well documented that people with acquired brain injuries can often pass traditional neuropsychological tests, yet still be socially impaired in their everyday lives (e.g., Bardenhagen et al., 1999; Shallice & Burgess, 1991).

These findings have led to the development of a variety of more naturalistic and advanced tasks to approximate the features and demands of everyday social functioning. Some studies have examined ability to identify mental states from videorecorded materials of social interactions. These have strengths in terms of ecological validity, but the materials are considerably more complex than first- and second-order false belief tasks both in terms of the length of the items and the multifaceted nature of the materials. Thus, Heavey, Phillips, Baron-Cohen, and Rutter (2000) developed the Awkward Moments Test, in which adults with AS/HFA watched videos of awkward social situations. Participants with AS/HFA were shown to be significantly more impaired at answering questions about the characters requiring mentalising abilities compared to healthy controls. In the Empathic Accuracy Task (Roeyers, Buysse, Ponnet, & Pichal, 2001), adolescents and adults with HFA had greater difficulty than controls when they viewed videos of naturally occurring conversations between two strangers and were then required to infer the characters’ unexpressed thoughts and feelings. In another study by Golan, Baron-Cohen, Hill, and Golan (2006), adults with AS/HFA were significantly poorer than controls at identifying the characters’ mental states from a set of alternatives from social scenes taken from films. Whilst the findings of these studies demonstrate that videorecorded materials are sensitive to picking up the deficits shown by those with AS/HFA, they have limited utility for pinpointing specific targets for intervention, in view of the wide range of potential sources of difficulty.

Other tasks developed to test subtle social deficits that are not detectable by more conventional tests of mentalising have focused on the examination of the ability to use and comprehend pragmatic language and communication, such as nonliteral utterances, sarcasm, irony, and humour. This type of social communication requires knowledge of what the speaker intends beyond a literal interpretation of the meaning of the words. Happé (1994) pioneered this type of research with the Strange Stories Test. Children and adults with autism, including a subset who passed both first- and second-order standard mentalising tasks, were presented with a series of everyday social vignettes where characters said things that they did not literally mean. All participants with ASD had difficulties in interpreting the characters’ mental states and understanding nonliteral utterances, and used fewer and more inappropriate mental state terms in their explanations. Subsequent studies using the same task have replicated this finding in those with autism and AS (Jolliffe & Baron-Cohen, 1999), and more recently in those with AS/HFA (Kaland et al., 2005). Using vignettes, cartoons, and jokes, Ozonoff and Miller (1996) reported that adults with HFA showed poorer understanding of humour, indirect requests, and inference compared to controls. A different set of social vignettes was developed by Kaland et al. (2002) to examine pragmatic understanding of a wide range of materials including lies, figures of speech, persuasion, irony, contrary emotions, empathy, and social blunders. Children and adolescents with AS were significantly more impaired at inferring the appropriate mental states and interpreting nonliteral utterances and behaviours compared to healthy controls, and needed more prompts and time to complete the tasks; this finding was recently replicated by Kaland, Mortensen, and Smith (2011). Although the finding that people with ASD have difficulties with nonliteral language appears robust, few studies have examined whether their difficulties with mentalising extend beyond situations with complex language demands. The present task extends previous work by including vignettes testing ability to infer the mental states that motivate human actions, in addition to mental states linked to nonliteral language.

Tasks assessing social and moral judgement have also highlighted difficulties in the identification and justification of inappropriate social behaviour in young people with autism. Thus, Baron-Cohen, O'Riordan, Stone, Jones, and Plaisted (1999) found that children with AS were significantly less able than controls to detect faux pas in stories where one of the characters made an embarrassing remark. When older samples were examined, adolescents with AS (Shamay-Tsoory, Tomer, Yaniv, & Aharon-Peretz, 2002) and also adults with AS (Zalla, Say, Stopin, Ahade, & Leboyer, 2009) were able to recognise the presence of faux pas, but failed to provide appropriate justifications of the characters’ behaviours or to explain the victims’ emotional reaction. A study by Loveland, Pearson, Tunali-Kotoski, Ortegon, and Gibbs (2001) showed that young people with autism were significantly more impaired than controls in judging and explaining inappropriate behaviour in simple social situations. In contrast, other studies have found social and moral judgements to be preserved in straightforward social situations in those with AS, but performance differences nevertheless emerged when multiple mentalistic factors needed to be taken into account in more complex social situations. For example, Grant, Boucher, Riggs, and Grayson (2005) reported that children with autism made similar judgements to controls in evaluating culpability on the basis of intention, and also judged injury to person more serious than damage to property, but were less able to justify their responses than controls. Shulman, Guberman, Shiling, and Bauminger (2012) also reported that young people with ASD gave more limited rationales than controls when asked to explain why certain behaviours were unacceptable (“that's bad”, “you can't do that”), and showed more utilitarian values oriented towards the negative consequences of the actions (e.g., “If I do that, the teacher will get mad”). Channon, Fitzpatrick, Drury, Taylor, and Lagnado (2010) found that individuals with AS tended to make more marked distinctions than controls between more valid and poorer justifications in their sympathy judgements for drivers causing car accidents. Similarly, those with AS were found to differentiated more markedly between intentional and unintentional human actions than controls in assigning blame for negative outcomes (Channon, Lagnado, Fitzpatrick, Drury, & Taylor, 2011). Moran et al. (2011) also examined intentionality, and concluded that individuals with HFA relied more on outcome information than on characters’ intentions when judging the moral permissibility of actions described in a series of vignettes. Taken together, these findings suggest that, when coping with difficult social situations, social reasoning in individuals with AS may be more rigid and rule based, compensating for inadequate social abilities through reliance on deliberate reasoning strategies drawn from social knowledge and conventions they have been explicitly taught or acquired through experience in the social world. This could explain why those with AS seem to have a more “black-and-white” approach to complex social situations requiring sophisticated emotional and social skills.

Evidence from more complex social reasoning tasks thus suggests that social judgement in those with ASD is often impoverished, and that childhood difficulties persist at least to some extent into adolescence and adulthood. It seems probable that incorrect understanding and interpretation of social behaviour will also lead to inappropriate responses in everyday social situations, but little experimental work has explored this directly. A study by Channon, Charman, Heap, Crawford, and Rios (2001) examined social problem solving in young people with AS, who were shown videos of awkward social situations and asked to generate and judge problem solutions. Those with AS were found to be impaired both in generating high-quality problem solutions, and in selecting optimal and preferred solutions; they differed most from those of the control group in the social appropriateness of their solutions. The present study investigated whether these difficulties were present in adults with AS, using a more comprehensive set of social cognition tasks developed by Channon and Crawford (2010); these materials have already been shown to be sensitive to impairments in everyday mentalising and social problem solving in participants with acquired brain injury. Three separate tasks, all involving scenarios describing real-life social situations, provide a means to assess multiple components of problem solving. The first task examines the ability to understand mentalistic language (sarcastic remarks), mentalistic actions, and physical events, both by explaining them and by selecting amongst alternatives. The second and third tasks assess ability to detect the awkward social elements in everyday problem situations, to generate appropriate solutions, and to select and judge the best way to resolve them. Taken together, the tasks therefore afforded the opportunity to determine the extent of any difficulties shown by adults with AS in generating responses, both when asked to make inferences to explain words and actions and to produce socially appropriate and effective problem solutions. The tasks also asked participants to select amongst alternative responses, to determine whether they could understand and solve social problems when they did not have to generate the solutions themselves.

## Methods

### Participants

Twenty-one participants (16 male, five female) who met DSM-IV^TR^ diagnostic criteria for AS (American Psychiatric Association, 2000) took part in the study. Inclusion criteria were fluency in English and age between 18 and 65. Those with any learning disability or history of illness of injury involving the brain were excluded. Comparison was made with a group of 21 healthy participants (16 male, five female) who also met the inclusion criteria and who matched those with AS in terms of age and years of education, consisting of five new individuals and 16 recruited previously to serve as controls for the acquired brain injury group described by Channon and Crawford (2010), using the same social cognition tasks. The groups did not differ significantly in age (AS mean = 40.00, *SD* = 14.93, control mean = 43.67, *SD* = 13.09), *t*(40) = 0.85, *p* = .402, IQ (Wechsler Test of Adult Reading: WTAR; Wechsler, 2001) (AS mean = 108.19, *SD* = 6.99, control mean = 108.40, *SD* = 5.52), *t*(39) = 0.10, *p* = .916, or years of education (AS mean = 14.86, *SD* = 1.59, control mean = 14.05, *SD* = 2.20), *t*(40) = 1.37, *p* = .180.

### Social cognition tests

The Mentalistic Interpretation, Social Problem Resolution, and Social Problem Fluency tasks described by Channon and Crawford (2010) were given to both groups. Each consisted of a series of written vignettes, and participants responded verbally.

#### Mentalistic interpretation task

This mentalising task included three types of items, with five of each type: sarcastic remarks, human actions, and physical events. For the mentalistic items (sarcasm and human actions) there was a brief description of the social context, ending with either a sarcastic remark or an action by one of the characters. The physical event items served as a control set, since they included a single character, but no reference to mental states was needed to account for the physical event. Participants were able to view the scenarios whilst answering the questions.

After reading the scenarios, participants were asked what the character meant by their sarcastic remark, why they carried out the action, or why the physical event happened. Clear correct explanations were scored 2, responses that were inadequate but not incorrect were scored 1, and responses that were incorrect or irrelevant were scored 0. After explaining the remarks, actions, or events, four alternative interpretations were presented. One of these provided a correct interpretation, an incorrect interpretation, an irrelevant interpretation, and one that was not necessarily incorrect but was much more general than the correct interpretation. Participants were asked to select the best alternative, and scored 1 or 0 for this. Total scores were also calculated for interpretation and selection of alternatives for the sarcastic items, action items, and averaged scores for the two types of items.

*Example of a sarcastic item.* “Liz and her friend often played tennis. Her friend always wanted to be best at everything. One day they were playing tennis in the local park. Liz knew that her friend expected to win the game. However, that day her friend did not win.

Liz said: ‘I suppose you'll say there's a hole in your racket!’”

*Question:* “What did Liz mean when she said that?”

*Example of an action item.* “Dave wanted to impress his new girlfriend Marie. He was cooking her a meal, but had never cooked before. Marie hoped it would be successful. Dave told her he had spent all day preparing it. When it came out of the oven it was badly burnt. Marie ate all her meal.

Afterwards she took a second helping of the food.”

*Question:* “Why did Marie take a second helping?”

*Example of a physical event item.* “Charlotte was sorting out her dirty washing. She put all the white clothes in one pile. Her new white dress had a bright red flower on it. She put it in the white clothes pile. Charlotte washed the white pile and took the clothes out of the washing machine.

All the white clothes had turned pink.”

*Question:* “Why did the white clothes turn pink?”

#### Social problem fluency task

This task consisted of 10 different brief social scenarios. It evaluated several aspects of social problem solving: sensitivity to awkwardness, capacity to generate a range of high quality solutions (solution fluency), and ability to select high quality solutions from alternatives. Participants were again able to view the scenarios whilst answering the questions.

*Awkwardness*. After reading each scenario, participants were asked to describe why it might be an awkward situation for the main character. Responses were scored 1 or 0, depending on whether they detected the awkward elements of the scenarios. They then rated awkwardness (not at all awkward = 0%; as awkward as it could possibly be = 100%).

#### Solution fluency and selection of alternatives

Participants were then given 1 minute to generate as many good ideas as they could to describe what they main character could do to deal with the situation. An example of an item is shown below. Responses were again judged on social sensitivity (S) and practical (P) effectiveness. For each scenario, two different optimal SP strategies were identified, where the solutions were both socially sensitive and practically effective. Two different S strategies that met only the socially sensitive criterion, and two different P strategies that met only the practically effective criterion were also identified. Responses that met neither criterion were classified as N. In order to ensure that fluency scores were not artificially inflated by the generation of multiple closely-related responses, a maximum score of one response per strategy was permitted. The maximum possible score for each scenario was thus 2 for each of the SP, S, and P strategies, and 1 for an N response.

Participants then selected the best solution from four alternatives for each scenario. Only one of the four possible solutions was both socially sensitive and practically effective (SP). The score consisted of the number of times that the SP alternative was selected as the optimal response.

*Example.* “Brian is on a train to work. Most people on the train are reading. The woman next to Brian keeps telling him about herself and then starts asking Brian personal questions.”

*Questions:* “Why might the situation be awkward for Brian?”; “How awkward a situation is it for Brian, out of 100%?”; “What could Brian do in this situation? Suggest as many good ideas as you can for dealing with the situation. You have one minute.”

#### Social problem resolution task

This task consisted of 10 brief scenarios describing awkward everyday social situations, to assess problem-solving ability. After reading each scenario, participants were asked to describe the best thing that the main character could do in the situation. Participants were able to view the scenarios whilst answering the questions.

Scoring of quality of solutions for each item took into consideration two dimensions, social sensitivity and practical effectiveness. Optimal solutions that were both socially sensitive and practically effective (SP) scored 2 points; solutions that were socially sensitive but not practically effective (S), or practically effective but not socially sensitive (P), scored 1 point; and solutions that were neither sensitive nor practical (N) scored 0. These scores were added for the 10 scenarios to give a quality of best solutions score. An example of an item is presented next.

*Example.* “Tony is always tired because he is kept awake by his new upstairs neighbours’ noisy dogs. The neighbours are very pleasant, but say that there is nothing they can do about the dogs.”

*Question:* “What is the best thing for Tony to do in this situation?”

### Procedure

Ethical permission for the study was granted by the Joint UCL/UCLH Committees on the Ethics of Human Research. All participants gave written informed consent for the study, and were given breaks between tasks as necessary, to avoid fatigue. All participants were given a brief interview, and then carried out the social cognition and executive tasks in counterbalanced order. Testing time was approximately one and a half to two hours, depending upon the individual.

## Results

Mean scores, standard deviations, and significance tests for scores are shown in Table 1. Nonparametric tests were used when scores were not normally distributed. A significance level of .05 was adopted throughout. Effect sizes for Cohen's d are also shown in Table 1.

**Table 1.** Mean scores and standard deviations for the social cognition measures.

	AS group		Control group			
	Mean	SD	Mean	SD	Significance	Effect size
Mentalistic interpretation task (%)						
Quality of interpretation						
Physical events	99.52	2.18	100.00	0.00	n/s	0.32
Sarcastic remarks	78.57	26.51	95.24	8.14	**	0.85
Mentalistic actions	87.62	15.46	96.67	4.83	*	0.79
Total interpretation score	83.10	19.90	95.95	3.75	**	0.90
Selection of best alternatives						
Physical events	98.10	6.02	100.00	0.00	n/s	0.45
Sarcastic remarks	80.95	24.88	96.19	10.24	*	0.80
Mentalistic actions	96.19	8.05	97.14	7.17	n/s	0.12
Total alternatives score	88.57	16.21	96.67	5.77	*	0.67
Selection of incorrect alternatives						
Physical events						
General alternative	1.90	6.02	0.00	0.00	n/s	0.45
Irrelevant alternative	0.00	0.00	0.00	0.00	.00	n/s
Wrong alternative	0.00	0.00	0.00	0.00	.00	n/s
Sarcastic remarks						
General alternative	6.67	13.17	2.86	9.56	n/s	0.00
Irrelevant alternative	1.90	6.02	0.00	0.00	n/s	0.45
Wrong alternative	10.48	18.57	0.95	4.36	*	0.71
Mentalistic actions						
General alternative	2.86	7.17	2.86	7.17	n/s	0.00
Irrelevant alternative	0.00	0.00	0.00	0.00	.00	n/s
Wrong alternative	0.95	4.36	0.00	0.00	n/s	0.31
Social problem resolution task (%)						
Quality of best solution	73.33	13.54	87.38	6.25	**	1.33
Social and practical (SP)	53.81	19.62	75.71	12.07	**	1.34
Social not practical (S)	18.10	14.01	10.00	8.37	*	0.70
Practical not social (P)	20.95	13.00	13.33	10.17	*	0.65
Neither social nor practical (N)	7.14	11.46	0.95	3.01	*	0.79
Social problem fluency task (%)						
Detection of awkwardness	91.43	13.52	97.14	5.61	n/s	0.55
Subjective awkwardness	64.23	12.14	63.19	13.61	n/s	0.08
Social and practical (SP)	58.57	12.26	67.86	10.07	*	0.83
Social not practical (S)	37.14	16.63	48.57	15.74	*	0.71
Practical not social (P)	30.95	15.13	37.62	18.41	n/s	0.40
Neither social nor practical (N)	23.33	23.52	24.76	20.15	n/s	0.07
Selection of best alternatives (SP)	80.48	20.85	81.90	11.67	n/s	0.08
Composite test scores (%)						
Generation score	65.16	10.94	79.84	6.87	**	1.61
Judgement score	84.52	15.64	89.29	5.76	n/s	0.41

Note: **p < .01, *p < .05.

### Mentalistic interpretation task

The two groups did not differ significantly in their quality of interpretation scores for the physical event items, *z* = 1.00, *p* = .317, and both groups showed ceiling effects. For the mentalistic items, participants with AS scored significantly below the control group both for sarcastic items, *t*(40) = 2.75, *p* = .009, and action items, *t*(40) = 2.56, *p* = .014; they also differed on total interpretation scores (sarcastic and action items combined), *t*(40) = 2.91, *p* = .006.

Selection amongst alternative answers was also examined. The groups did not differ significantly on the physical event items, *z* = 1.43, *p* = .152, nor on the action items, *t*(40) = 0.41, *p* = .688; both groups approached ceiling levels of performance. However, those with AS chose significantly fewer correct alternatives for the sarcastic items, *t*(40) = 2.60, *p* = .013; they chose more incorrect, irrelevant, and general alternatives than the control group. Examination of the alternatives for the sarcastic items showed that those with AS selected more of each type of alternative, but the group difference reached significance only for the incorrect category, *t*(40) = 2.29, *p* = .027. The groups differed significantly on total selection of alternatives scores (sarcastic and action items combined), *t*(40) = 2.16, *p* = .037.

### Social problem fluency task

The groups were compared on scores for whether they were able to identify the awkward elements of the situations, and the difference was marginally significant, *z* = 1.92, *p* = .055; the participants with AS scored below the control group. The two groups did not differ significantly in their subjective ratings of the how awkward the situations were for the main character, *t*(40) = 0.26, *p* = .796.

When the number of optimal (SP) solutions generated for the scenarios was examined, those with AS produced significantly fewer SP and S solutions than the control group: SP, *t*(40) = 2.68, *p* = .011; S, *t*(40) = 2.29, *p* = .028. The groups did not differ in the number of P and N solutions produced: P, *t*(40) = 1.28, *p* = .207; N, *t*(40) = 0.21, *p* = .834. When selection amongst alternative answers was examined, they did not differ in the number of SP alternatives chosen as the best response, *t*(40) = 0.27, *p* = .786.

### Social problem resolution task

Comparison of the two groups on the social problem resolution task showed that the overall quality of the solutions generated by those with AS was significantly lower than the solutions generated by the control group, *t*(40) = 4.32, *p* = .0001. Examination of the pattern of responses showed that those with AS gave fewer optimal (SP) solutions than the control group, *t*(40) = 4.36, *p* = .0001, and gave more S responses, *t*(40) = 2.27, *p* = .028, more P responses, *t*(40) = 2.12, *p* = .041, and more N responses, *t*(40) = 2.39, *p* = .021.

### Composite test scores

Two composite scores, generation and judgement, were derived from the key measures in each of the three tasks. The generation score was calculated by averaging the total interpretation score from the mentalistic interpretations task, the number of optimal solutions generated for the solution problem fluency task, and the number of optimal solutions generated for the solution problem resolution task. The groups differed significantly in generation scores, *t*(40) = 5.21, *p* = .0001. Similarly, a judgement score was calculated by averaging the total selection of alternatives score from the mentalistic interpretations task and the selection of alternatives score from the solution problem fluency task. The groups did not differ significantly in judgement scores, *t*(40) = 1.31, *p* = .198. Using a cut off of 70%, 2 of the control group and 15 of the AS group fell at or below this for the generation score; for the judgement score, none of the control group and 4 of the AS group fell at or below 70%.

## Discussion

This study was designed to examine understanding and interpretation of social behaviour, and the extent of any difficulties in social judgement shown by adults with AS on a set of social cognition tasks based on awkward everyday scenarios. The mentalistic interpretations task assessed ability to make inferences to interpret sarcastic remarks and human actions, and also control physical events; the social problem fluency task and the social problem resolution task assessed ability to generate and judge problem solutions. Those with AS thus performed significantly below the control group for many of the social cognition measures that involved generating their own interpretations and problem solutions, and this was reflected in the overall generation composite scores; the groups did not differ overall on the composite judgement scores, although one of the two component measures (mentalistic interpretations) did show a difference.

For the mentalistic interpretations task, the interpretations generated by those with AS were significantly lower in quality than those of the control group, both for sarcastic remarks and actions. Those with AS also made poorer choices than the control group for the sarcastic remarks but not for the human actions when asked to select amongst alternative interpretations. The groups did not differ on the control physical event scenarios for either generation or judging alternatives. For the social problem fluency task, those with AS had greater difficulty in detecting the awkward elements of the problem situations, although there was no difference between groups in their subjective ratings of perceived awkwardness for the main characters. Solution fluency was poorer for the participants with AS, since they generated fewer optimal solutions (those that were both socially sensitive and practically effective) and also fewer solutions that were socially sensitive but not practically effective; the groups did not differ in the number of solutions generated that were practical but not socially sensitive, or solutions that met neither criterion. By contrast, the groups did not differ significantly in the number of optimal solutions selected when asked to judge sets of alternative solutions. The sample sizes in the present study were relatively small, and this may have limited the potential to detect differences when judging between alternatives both for the interpretation of human actions and for social problem solutions. However, inspection of the mean scores suggests that there were ceiling effects for both groups on some measures; some of the items may therefore have been too easy to detect subtle difficulties in a relatively high-functioning clinical group such as those with AS.

It is well-established that mentalising skills are deficient in people with AS (e.g., Frith, 2001), leading to impaired social and emotional skills. These impairments are likely to contribute to the weaknesses in performance shown on higher-level social cognition tasks such as the ones described earlier. How such impairments in emotional and social skills translate into everyday behaviour is, however, less well-understood; this was the focus of the present study. Different aspects of social performance were examined using scenarios describing everyday social situations.

The mentalistic interpretations task required participants to make contextual interpretations of people's motivations through their words or actions. Previous studies have shown that individuals with AS have difficulties with interpreting complex emotions from video clips (Golan et al., 2006; Heavey et al., 2000), and from vignettes in which nonliteral inferences must be made (e.g., Happé, 1994; Kaland et al., 2002, 2005, 2011). The present study used written vignettes in order to remove nonverbal cues, for example tone of voice and facial expression, such that it was necessary for participants to use contextual cues to detect conflict between the literal meaning of the words and the social context. Consistent with previous findings, those with AS were less able to interpret sarcasm than were control participants, even when presented with alternative meanings to aid performance. The current findings extend previous work by demonstrating that difficulties in making use of contextual cues were found to encompass the interpretation of actions as well as words, since those with AS were also impaired in working out the significance of people's behaviour in different social contexts. Difficulty in understanding the intentions behind everyday actions implies social disadvantage on a broader scale than impairment confined to the meanings of language alone. The same pattern of impairments has been reported in individuals with focal damage to frontal brain regions thought to underpin emotional and social processing (Channon et al., 2007).

In the social problem fluency task, participants with AS tended to show reduced ability to describe the awkward aspects of social situations when asked to identify these for the main character of the scenario. This may reflect specific difficulties in putting themselves in the shoes of the story characters. Similarly, impoverished fluency in generating a range of solutions that were both socially appropriate and practically effective may also be explained by a deficit in mentalising, as careful appreciation of social problems ideally necessitates consideration of the situation from the perspective of all the relevant participants. This finding is consistent with previous research showing that adolescents and adults with AS find it more difficult than healthy controls to explain behaviour that is generally seen to be socially inappropriate (Baron-Cohen et al., 1999; Loveland et al., 2001). Performance on the social problem resolution task extended these findings by showing that the adults with AS also generated less satisfactory “best” solutions for a set of problem situations; they again had greater difficulty in finding solutions that were socially appropriate as well as practically effective. This supports a previous study by Channon et al. (2001), where adolescents with AS were found to be more impaired than controls in both generating and selecting high-quality and socially appropriate problem solutions. Limited appreciation of the social appropriateness of different courses of action is another potential indication of perspective-taking difficulties in evaluating scenarios from the viewpoints of each of the characters.

The present set of tasks permitted the identification of social impairments in those with AS by presenting them with a series of social scenarios. These scenarios were brief and close to real life to be as naturalistic as possible and to reduce the need for imagination. Performance was examined from a number of different angles that included explaining the meaning of people's words and actions, choosing a preferred response from a set of alternatives, explaining the awkwardness underlying the different social situations, and generating appropriate solutions, which were then systematically examined and categorised. This type of analysis provides insight into the nature of reasoning processes and associated errors in those with AS. It therefore allows us to examine how deficits in mentalising affect social behaviour and strategies in everyday life, highlighting in what ways and circumstances individuals with AS can appear “awkward” or socially inappropriate, thereby hindering the formation of positive social relationships.

Not all aspects of performance were found to be impaired in those with AS. Thus, there was no difference between the groups when they rated the level of perceived awkwardness in the social problem fluency task, implying that participants with AS may be able to identify situations or situational elements that are considered awkward by the general population. A potential implication of this finding is that they may have acquired such knowledge through everyday experience in the social world. Zalla et al. (2009) suggested that age might be a factor in explaining why adults with AS in their own study were able to use compensatory strategies to carry out social judgements such as identifying faux pas (although they still failed to justify their responses), whereas children with AS (Baron-Cohen et al., 1999) were not able to detect faux pas accurately. This has parallels with the finding from the present study that participants with AS found it more difficult to identify the awkward elements in those same problem situations, and the solutions they generated in response were also poorer than those of controls. This suggests that although they were able to recognise awkward situations, individuals with AS did not truly understand why they were awkward. Similarly, in a study looking at the recognition of embarrassment in social scenarios, Hillier and Allinson (2002) found that children with HFA were able to recognise the influence of the presence of an audience and also the type of audience, to rate the character's level of embarrassment, and to justify their ratings, although their explanations were sometimes less accurate than those of controls. As a potential explanation of these findings, the authors suggested that participants with HFA had learned to identify cues linked with the emotion of embarrassment, and that they were able to pick up on these cues in the scenarios to make their ratings and justifications, thereby using cognitive strategies to circumvent the need for empathic skills. This explanation could also account for the absence of differences in awkwardness ratings between groups in the present study; individuals with AS may have made simple judgements of this nature on the social knowledge and social conventions they had explicitly learnt through experience. Studies examining social reasoning in individuals with AS has shown that their thinking can in fact often appear more rule based and “black and white” compared to healthy individuals, especially when they are asked to reason about more complex social situations requiring the appreciation of multiple social and emotional factors (Channon et al., 2010, 2011; Grant et al., 2005; Moran et al., 2011; Shulman et al., 2012). Thus, reliance on social expectations and conventions when evaluating or responding to awkward situations such as in the present study could lead to a discrepancy between knowledge that a situation is conventionally seen as appropriate versus inappropriate, and comprehension of the reasons behind this. In a clinical context, this measure could potentially be refined to identify such discrepancies and to target intervention strategies. This could be geared either towards expanding the individual's knowledge of social events (if they do not perceive the situations as awkward), or expanding their knowledge of potential explanations for this awkwardness, albeit at a cognitive-compensatory rather than automatic-emotional level.

A rule-based approach to social judgements could also be put forward to explain the absence of difference in performance between the two groups in the current study when they had to choose an explanation among alternatives for the human actions described in the mentalistic interpretations scenarios and when they could choose solutions to social problems in the social problem fluency task. Identifying the correct answer amongst a set of alternatives is less cognitively demanding than generating an appropriate interpretation or strategy. Thus, participants with AS might have found it easier to apply their knowledge of social expectations and conventions to identify the most appropriate answer. However, participants with AS were impaired relative to controls in selecting the correct explanations from sets of alternatives for the sarcastic items. Identification and comprehension of sarcasm may therefore represent a more stringent and sophisticated test of mentalising, requiring people to appreciate that the speaker does not intend the meaning of the words to be taken literally; interpretation of the correct meaning depends on the social context.

The present findings show that the tasks used in this study, which have already been found to be sensitive to adult-acquired brain injury (Channon & Crawford, 2010), also appear to be sensitive to the difficulties of those with AS in generating appropriate social interpretations and problem solutions, and may thus prove a potentially useful clinical tool for profiling an individual's strengths and weaknesses in everyday social functioning. These measures provide information about difficulties in the spontaneous processing of social information through open-ended verbal responses. The tasks also assess capacity to judge the quality of alternative responses when the need to generate ideas is removed, although this component of social problem-solving showed ceiling effects in the present study and therefore proved less revealing for participants with AS than for those with adult-acquired brain injury. Whilst considerable efforts have been devoted to improving social functioning in ASD, particularly with children at the more severe end of the spectrum, relatively few studies have addressed this concern in adults who are high functioning. Moreover, little is known about which components underpinning social skill are relatively sensitive or insensitive to change (see, e.g., Mueser & Bellack, 2007; Schreiber, 2011). Intervention programmes have been shown to suffer from a lack of generalisability in the transfer of skills from therapeutic to real-life settings (Cappadocia & Weiss, 2010; Parsons & Mitchell, 2002). This may be partially attributable to reliance upon teaching broad, nonspecific social abilities, such as conversational skills and appropriate nonverbal behaviours (Rao, Beidel, & Murray, 2008). The identification of specific deficits in aspects of performance using tasks such as those described in this study could be used to tailor individual programmes for intervention, to evaluate whether this enhances performance. These tools could therefore be used to extend training programmes designed to improve social skills. Together with other recent findings, this work potentially provides a basis for the development of interventions to teach those with AS to think and reason about social problems and situations more flexibly, especially in situations where mere application of “social rules” and abstract cognitive skills is insufficient to generate judgements that require a more comprehensive appreciation of the complexity of human behaviour.
